# Supplementary figures and images for: Genetic evidence that uptake of the fluorescent analog 2NBDG occurs independently of known glucose transporters
Source: PLoS One. 2022 Aug 24;17(8):e0261801. doi: 10.1371/journal.pone.0261801 (PMC9401136; doi:10.1371/journal.pone.0261801)

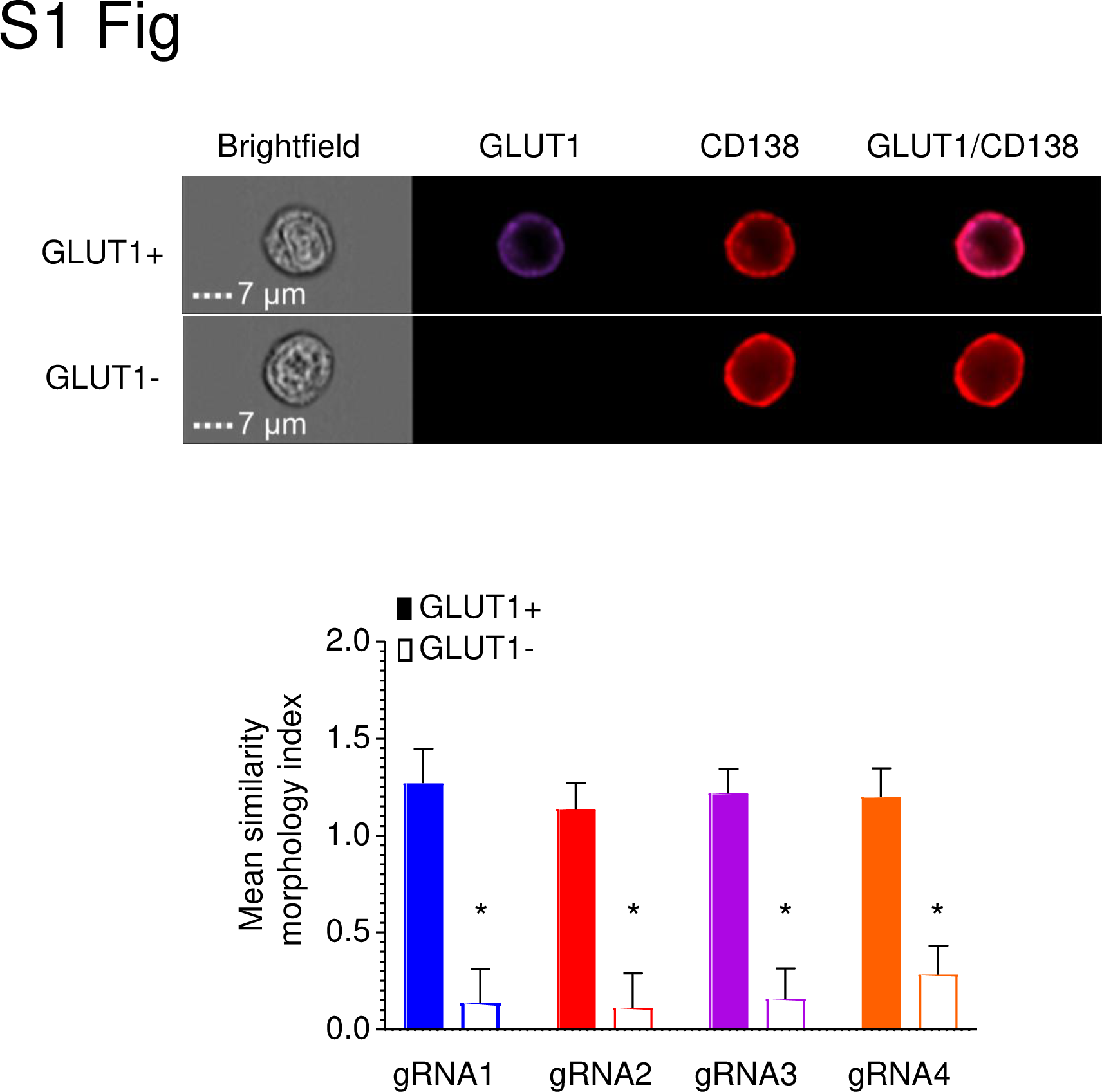

Supplement: S1 Fig — Cells transduced with Slc2a1 gRNA were stained for GLUT1 and CD138. (A) Representative images from GLUT1-sufficient (top) and GLUT1-deficient cells (bottom) in the same culture. (B) Quantification of mean similarity morphology indices for GLUT1 and CD138 in GLUT1+ (filled bars) and GLUT1- (hollow bars) cells. Pooled data from three independent experiments. *p<0.05 by Šídák’s multiple comparisons test. (TIF) [file pone.0261801.s001.tif]

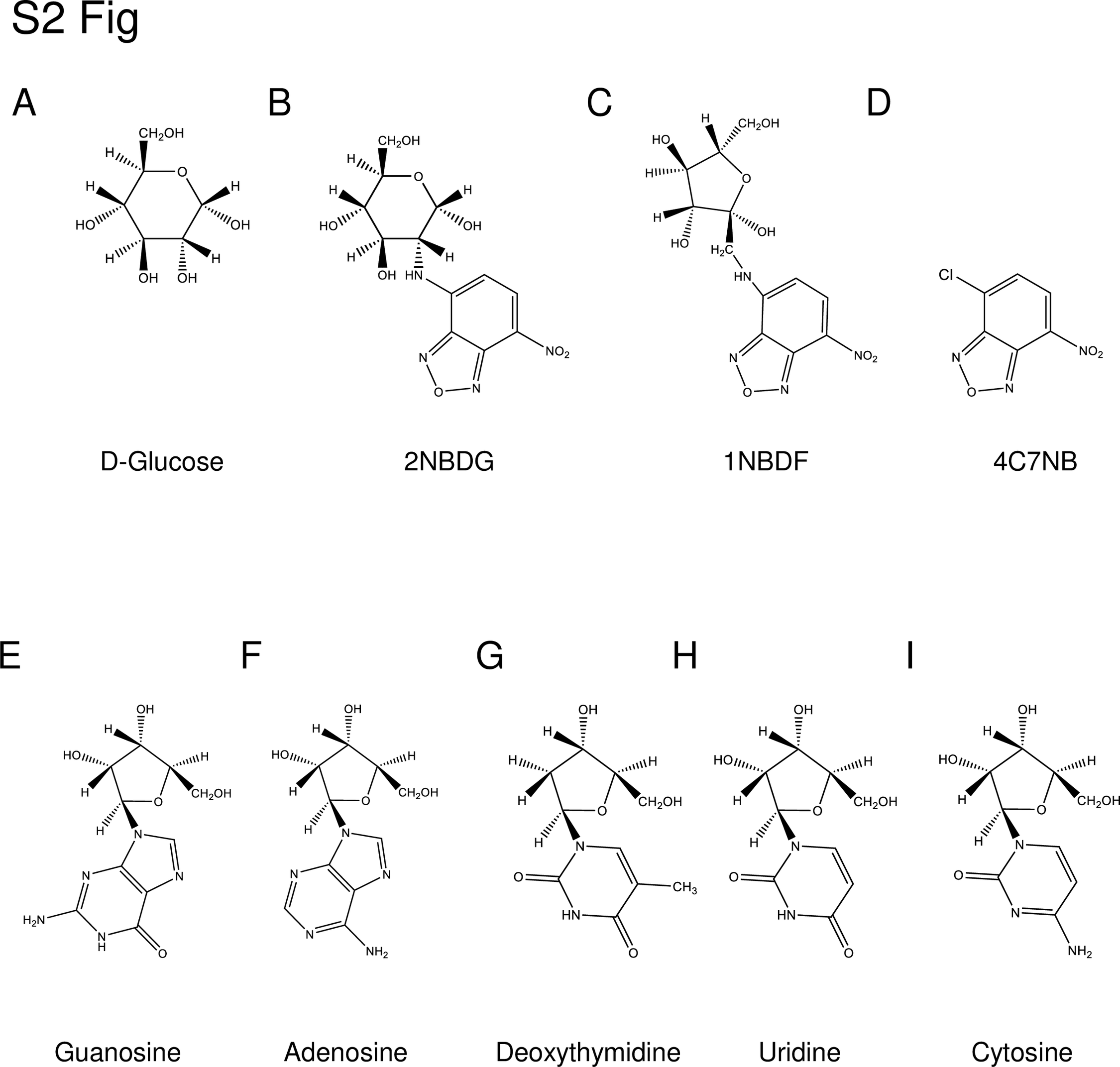

Supplement: S2 Fig — Chemical structures of (A) D-Glucose, (B) 2NBDG, (C) 1NBDF, (D) 4C7NB, (E) Guanosine, (F) Adenosine, (G) Deoxythymidine, (H) Uridine, and (I) Cytosine. Structures generated using ChemDraw v.20.1.1. (TIF) [file pone.0261801.s002.tif]

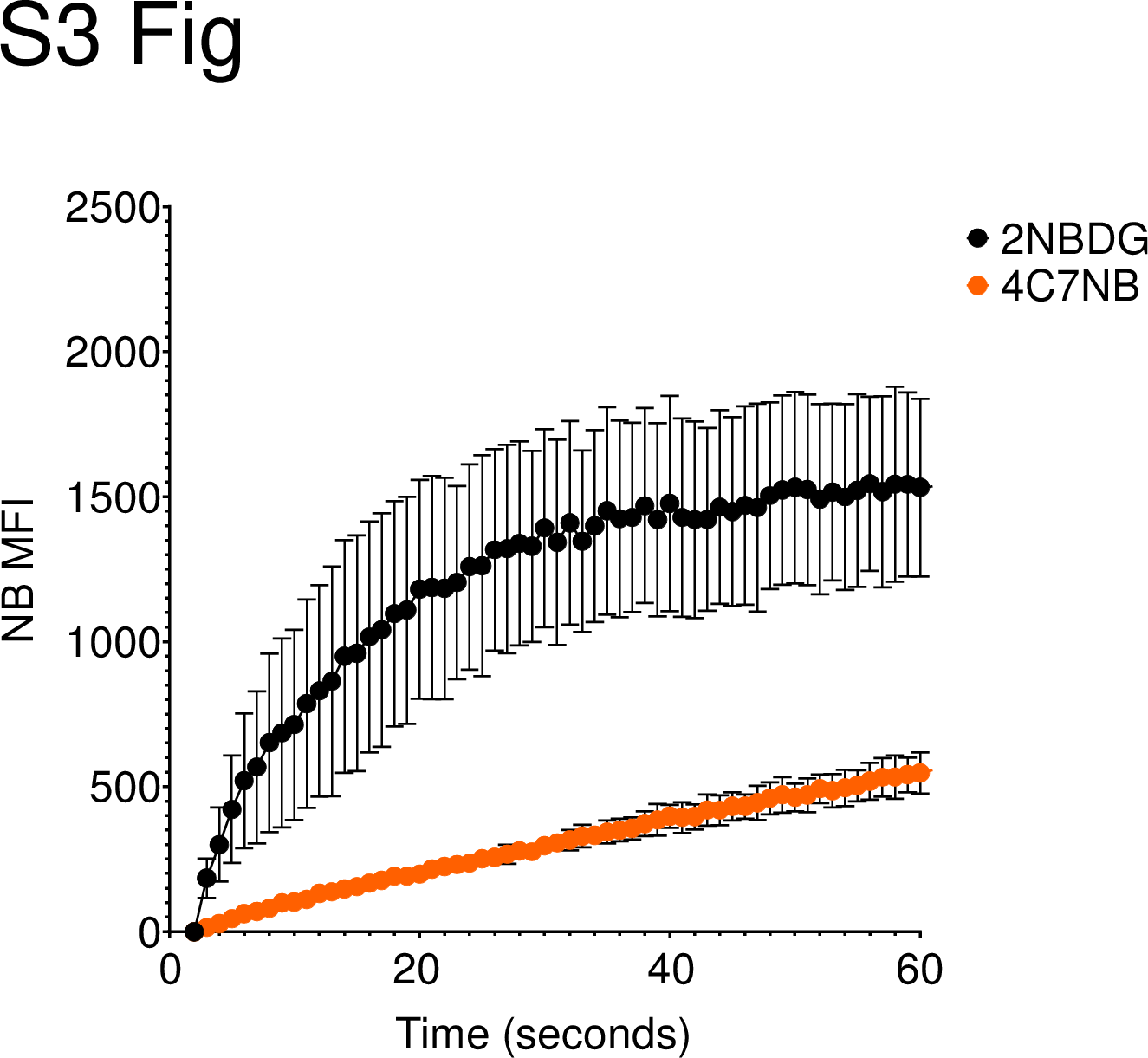

Supplement: S3 Fig — (TIF) [file pone.0261801.s003.tif]

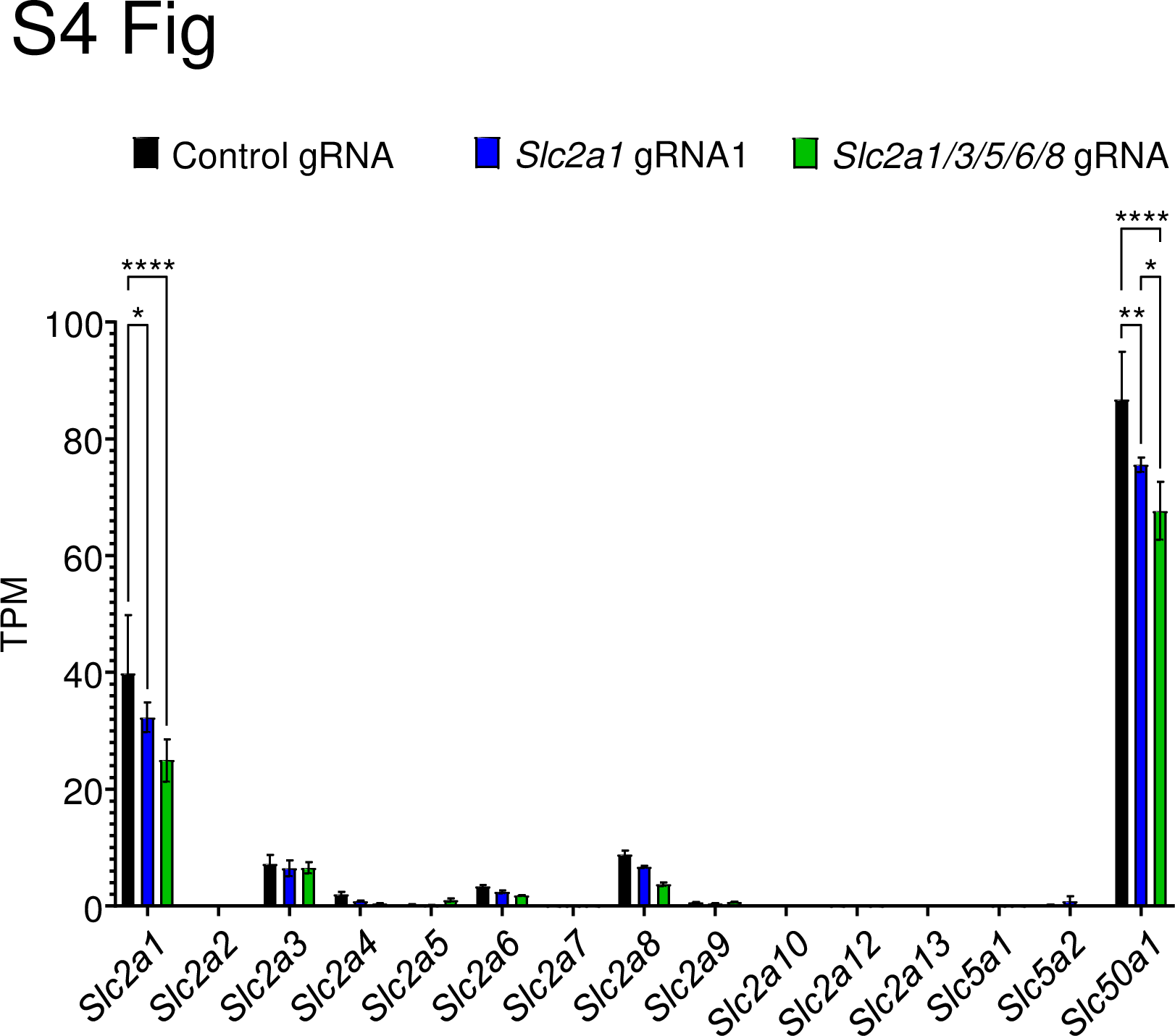

Supplement: S4 Fig — RNA-Seq analysis of sugar transporter transcript levels in control gRNA-transduced (black), Slc2a1 gRNA-transduced (blue), and Slc2a1/3/5/6/8 gRNA-transduced (green) 5TGM1-Cas9 cultures. Three biological replicates were analyzed for each population and data is represented as mean values +/- SEM. *p<0.05, **p<0.01, and ***p<0.001 by paired two-way ANOVA. (TIF) [file pone.0261801.s004.tif]
