# Supplementary material for: Genetic evidence that uptake of the fluorescent analog 2NBDG occurs independently of known glucose transporters
Source: PLoS One. 2022 Aug 24;17(8):e0261801. doi: 10.1371/journal.pone.0261801 (PMC9401136; doi:10.1371/journal.pone.0261801)
Supplement: Attachment — Submitted filename: Manuscript review 2-NBDG.docx [file pone.0261801.s005.docx]

Manuscript review:

Title: **Genetic evidence that uptake of the fluorescent analog 2NBDG occurs independently of known glucose transporters**

Manuscript: PONE-D-21-38751

Corresponding Author: Deepta Bhattacharya

**Summary:** Authors utilize CRISPER-Cas 9 gene technology to ablate GLUT1 and show that ^14^C-glucose uptake is reduced, but that the uptake of 2-NBDG, a common fluorescent glucose that has widespread use in glucose uptake studies, is not affected. In addition, the uptake of 2-NBDG is not affected by knock out of other glucose transporters, or by ablation of select nucleoside, nucleotide, or ABC transporters. Authors conclude that 2-NBDG is taken up by cells by an unknown mechanism, but independent of glucose specificity.

**Critique summary:** The methodology and experimental design are appropriate, well reported and clearly described. The genetic editing technique employed in this study is a unique approach to modulate glucose transporters. The results of this study support other published work and call into question the efficacy of using 2-NBDG as a surrogate for glucose in glucose uptake studies. The data demonstrating that GLUT1 does not transport 2-NBDG is better documented than the case for the non-involvement of the other transporters studied (see below). Given the widespread use of this analog in the research literature, it is important that these results are published.

**Questions and concerns:**

1) I have some questions about the controls utilized in this study. The ablation of GLUT1 (Fig 1) is confirmed by both protein analysis (GLUT1 immunostaining) and by functional analysis (^14^C-glucose uptake). However, neither protein analysis nor functional analysis is utilized to confirm the knock out of the other putative transporters of 2-NBDG. Rather the authors rely on DNA sequencing to show the ablation of the targeted transporter and those analysis all show some unmodified targets. The actual loss of the receptor is not demonstrated. In fact, the ablation of GUT1 actually does not completely knock out GLUT1 (Fig 1), so why would we expect a complete ablation of the other transporters. I do find that data demonstrating no increase in 2NBDG negative cells convincing that the receptor was not involved in transport. (I would have expected that a certain population of cells would have both alleles ablated and thus, if that receptor were involved in transport, those cells should show up as 2NBDG negative.). Please comment on why receptor analysis and function are not reported for transporters other than GLUT1.
2) I would revise the conclusion that ‘2NBDG is actively transported’ (line 66 of introduction). ‘Active’ implies energy input required (eg ATP) for which no evidence is provided. Also, I am not entirely convinced that the data can distinguish between an actual transport process as opposed to a binding and internalization via protein recycling. The 1NBDGF control does demonstrate mediated uptake, but not the mechanism. It would have been interesting to measure uptake of just the chromophore (NBD), which I expect would be lipid soluble. The role of the fluorescent chromophore in regulating uptake is not clear.
3) Please define TPM and MFI in figure legends. Also, while stated in the figure legend, Fig 2B would be clearer if the spleen cells and bone marrow cells were designated on the figure itself (eg white print on the black photos). This is just a suggestion.
