# Supplementary material for: Genetic evidence that uptake of the fluorescent analog 2NBDG occurs independently of known glucose transporters
Source: PLoS One. 2022 Aug 24;17(8):e0261801. doi: 10.1371/journal.pone.0261801 (PMC9401136; doi:10.1371/journal.pone.0261801)
Supplement: Attachment — Submitted filename: Response to reviewers-v5_db.docx [file pone.0261801.s007.docx]

Journal Requirements:

Please review your reference list to ensure that it is complete and correct. If you have cited papers that have been retracted, please include the rationale for doing so in the manuscript text, or remove these references and replace them with relevant current references. Any changes to the reference list should be mentioned in the rebuttal letter that accompanies your revised manuscript. If you need to cite a retracted article, indicate the article’s retracted status in the References list and also include a citation and full reference for the retraction notice.

We have examined all the references and found them to be complete and correct. All cited manuscripts are currently accepted and available on the publisher’s website or on PubMed Central.

[Note: HTML markup is below. Please do not edit.]

Reviewers' comments:

Reviewer's Responses to Questions

**Comments to the Author**

1. If the authors have adequately addressed your comments raised in a previous round of review and you feel that this manuscript is now acceptable for publication, you may indicate that here to bypass the “Comments to the Author” section, enter your conflict of interest statement in the “Confidential to Editor” section, and submit your "Accept" recommendation.

Reviewer #2: All comments have been addressed

Reviewer #3: (No Response)

2. Is the manuscript technically sound, and do the data support the conclusions?

The manuscript must describe a technically sound piece of scientific research with data that supports the conclusions. Experiments must have been conducted rigorously, with appropriate controls, replication, and sample sizes. The conclusions must be drawn appropriately based on the data presented.

Reviewer #2: (No Response)

Reviewer #3: Yes

3. Has the statistical analysis been performed appropriately and rigorously?

Reviewer #2: (No Response)

Reviewer #3: Yes

4. Have the authors made all data underlying the findings in their manuscript fully available?

The [PLOS Data policy](http://www.plosone.org/static/policies.action#sharing) requires authors to make all data underlying the findings described in their manuscript fully available without restriction, with rare exception (please refer to the Data Availability Statement in the manuscript PDF file). The data should be provided as part of the manuscript or its supporting information, or deposited to a public repository. For example, in addition to summary statistics, the data points behind means, medians and variance measures should be available. If there are restrictions on publicly sharing data—e.g. participant privacy or use of data from a third party—those must be specified.

Reviewer #2: (No Response)

Reviewer #3: Yes

5. Is the manuscript presented in an intelligible fashion and written in standard English?

PLOS ONE does not copyedit accepted manuscripts, so the language in submitted articles must be clear, correct, and unambiguous. Any typographical or grammatical errors should be corrected at revision, so please note any specific errors here.

Reviewer #2: (No Response)

Reviewer #3: Yes

6. Review Comments to the Author

Please use the space provided to explain your answers to the questions above. You may also include additional comments for the author, including concerns about dual publication, research ethics, or publication ethics. (Please upload your review as an attachment if it exceeds 20,000 characters)

Reviewer #2: (No Response)

Reviewer #3: Authors have made a considerable revision to the original manuscript. My concern is that they generalized results obtained from a limited types of cells expressing GLUT1 as "Thus, cellular uptake of 2NBDG is not a faithful indicator of glucose transport ...” (the last sentence in Abstract). I would strongly recommend authors to confine their statement to glucose transport at least through GLUT1, since no experiment was done for cells intrinsically expressing such as GLUT2.

We apologize for our obstinacy, but even for GLUT1, we cannot conclude with certainty that 2NBDG cannot be transported through it at all. Instead, the data show that there are other glucose transporter-independent pathways by which 2NBDG predominantly enters cells. This background is what makes 2NBDG unreliable as a surrogate for glucose uptake. Whether GLUT2 or other glucose transporters are expressed does not change this fact. In this sense, we feel that it would be an overreach to adopt this suggestion and claim 2NBDG cannot be transported by GLUT1.

7. PLOS authors have the option to publish the peer review history of their article ([what does this mean?](https://journals.plos.org/plosone/s/editorial-and-peer-review-process#loc-peer-review-history)). If published, this will include your full peer review and any attached files.


If you choose “no”, your identity will remain anonymous but your review may still be made public.


**Do you want your identity to be public for this peer review?** For information about this choice, including consent withdrawal, please see our [Privacy Policy](https://www.plos.org/privacy-policy).

Reviewer #2: No

Reviewer #3: No
