# Supplementary material for: Genetic evidence that uptake of the fluorescent analog 2NBDG occurs independently of known glucose transporters
Source: PLoS One. 2022 Aug 24;17(8):e0261801. doi: 10.1371/journal.pone.0261801 (PMC9401136; doi:10.1371/journal.pone.0261801)
Supplement: Attachment — Submitted filename: Response to reviewers-v5.docx [file pone.0261801.s008.docx]

**Comments to the Author**

1. If the authors have adequately addressed your comments raised in a previous round of review and you feel that this manuscript is now acceptable for publication, you may indicate that here to bypass the “Comments to the Author” section, enter your conflict of interest statement in the “Confidential to Editor” section, and submit your "Accept" recommendation.

Reviewer #3: All comments have been addressed

2. Is the manuscript technically sound, and do the data support the conclusions?

The manuscript must describe a technically sound piece of scientific research with data that supports the conclusions. Experiments must have been conducted rigorously, with appropriate controls, replication, and sample sizes. The conclusions must be drawn appropriately based on the data presented.

Reviewer #3: Partly

3. Has the statistical analysis been performed appropriately and rigorously?

Reviewer #3: Yes

4. Have the authors made all data underlying the findings in their manuscript fully available?

The [PLOS Data policy](http://www.plosone.org/static/policies.action#sharing) requires authors to make all data underlying the findings described in their manuscript fully available without restriction, with rare exception (please refer to the Data Availability Statement in the manuscript PDF file). The data should be provided as part of the manuscript or its supporting information, or deposited to a public repository. For example, in addition to summary statistics, the data points behind means, medians and variance measures should be available. If there are restrictions on publicly sharing data—e.g. participant privacy or use of data from a third party—those must be specified.

Reviewer #3: Yes

5. Is the manuscript presented in an intelligible fashion and written in standard English?

PLOS ONE does not copyedit accepted manuscripts, so the language in submitted articles must be clear, correct, and unambiguous. Any typographical or grammatical errors should be corrected at revision, so please note any specific errors here.

Reviewer #3: Yes

6. Review Comments to the Author

Please use the space provided to explain your answers to the questions above. You may also include additional comments for the author, including concerns about dual publication, research ethics, or publication ethics. (Please upload your review as an attachment if it exceeds 20,000 characters)

Reviewer #3: This reviewer agrees an involvement of non-GLUT1-mediated mechanism in the uptake of 2-NBDG into certain cell types. In this respect, this manuscript provides valuable information to the scientific community. However, this reviewer unfortunately can not accept authors' conclusion and the title in the present form. Authors' evidence was obtained from limited cell types and was limited to GLUT1. However, the title of the manuscript included sentence "uptake of the fluorescent analog 2NBDG occurs independently of 'known' glucose transporters". This title would not be adequate, if 2-NBDG uptake occurs through GLUT2 in other cell lines to a significant extent. To greatly increase values of this important manuscript, this reviewer recommends authors to refine the title and the conclusive sentence, or at least to mention GLUT2.

We have modified the abstract to now end with the statement, “Thus, cellular uptake of 2NBDG is not necessarily a faithful indicator of glucose transport and is promoted by an unknown mechanism.” Further, the discussion now concludes the manuscript with the statement, “… we strongly advise that despite their convenience, these assays should not be considered as direct indicators of glucose uptake.”

7. PLOS authors have the option to publish the peer review history of their article ([what does this mean?](https://journals.plos.org/plosone/s/editorial-and-peer-review-process#loc-peer-review-history)). If published, this will include your full peer review and any attached files.


If you choose “no”, your identity will remain anonymous but your review may still be made public.


**Do you want your identity to be public for this peer review?** For information about this choice, including consent withdrawal, please see our [Privacy Policy](https://www.plos.org/privacy-policy).

Reviewer #3: No
